# Personalizing Breast Cancer Surgery: Harnessing the Power of ROME (Radiological and Oncoplastic Multidisciplinary Evaluation)

**DOI:** 10.3390/jpm15030114

**Published:** 2025-03-14

**Authors:** Liliana Barone Adesi, Marzia Salgarello, Alba Di Leone, Giuseppe Visconti, Marco Conti, Paolo Belli, Lorenzo Scardina, Giulio Tarantino, Gianluca Franceschini

**Affiliations:** 1 Department of Plastic, Reconstructive and Esthetic Surgery, Department of Science and Health of Women, Children and Public Health, Fondazione Policlinico Universitario Agostino Gemelli IRCCS, 00168 Rome, Italy; liliana.baroneadesi@policlinicogemelli.it (L.B.A.); marzia.salgarello@policlinicogemelli.it (M.S.); giuseppe.visconti@policlinicogemelli.it (G.V.); 2 Multidisciplinary Breast Center, Department of Science and Health of Women, Children and Public Health, Fondazione Policlinico Universitario Agostino Gemelli IRCCS, 00168 Rome, Italy; alba.dileone@policlinicogemelli.it (A.D.L.); lorenzoscardina@libero.it (L.S.); gianlucafranceschini70@gmail.com (G.F.); 3 Department of Thoracic and Cardiovascular Radiology, Fondazione Policlinico Universitario Agostino Gemelli IRCCS, 00168 Rome, Italy; marco.conti@policlinicogemelli.it (M.C.); paolo.belli@policlinicogemelli.it (P.B.)

**Keywords:** breast cancer, personalized treatment, multidisciplinary team, breast surgery, oncological and aesthetic outcomes

## Abstract

Breast cancer treatment has evolved significantly in recent decades, with personalized care models gaining prominence both for the optimization of oncological outcomes and aesthetic results. At the Fondazione Policlinico Universitario Agostino Gemelli IRCCS in Rome, Italy, we have developed a multidisciplinary, evidence-based model for the management of breast cancer patients, called ROME (Radiological and Oncoplastic Multidisciplinary Evaluation). This innovative model integrates the expertise of various specialists in a seamless, patient-centered approach to improve treatment planning and outcomes. ROME involves a collaborative framework between radiologists, oncologists, surgeons, pathologists, oncoplastic specialists and psychologists. The process begins with the detailed radiological evaluation of tumors using advanced imaging techniques, which is then complemented by an oncoplastic assessment to evaluate potential surgical approaches that ensure optimal oncological resections while preserving or enhancing breast aesthetics. The combination of these evaluations allows the team to tailor treatment plans according to the patient’s specific clinical profile, including tumor characteristics, genetic factors, and aesthetic considerations. A key feature of the ROME model is the continuous integration of evidence-based guidelines with real-time multidisciplinary input. This enables the personalization of surgical strategies, ensuring that each patient receives a treatment plan that balances the need for effective cancer control with the desire for an optimal aesthetic result. Since its implementation, ROME has demonstrated significant improvements in both oncological and cosmetic outcomes, leading to enhanced patient satisfaction and quality of life. The success of ROME underscores the importance of a holistic and collaborative approach to breast cancer treatment, one that integrates clinical, radiological, and aesthetic perspectives to offer a truly personalized and patient-focused care experience. As evidence continues to accumulate, ROME stands as a model for personalized breast cancer surgery, setting a new standard for care in multidisciplinary oncology settings.

## 1. Introduction

The treatment of breast cancer has evolved significantly over the past decades, with an increasing emphasis on personalized care models that optimize both oncologic and cosmetic outcomes [1,2]. Traditionally, the focus of breast cancer treatment has been on achieving optimal oncologic control, often with little regard for the aesthetic and psychological well-being of patients. However, with increasing awareness of the importance of an integrated and holistic approach to patient care, personalized treatment strategies have become increasingly important [3]. These approaches not only prioritize cancer removal, but also aim to preserve or improve quality of life, particularly in terms of body image and emotional recovery.

At the “Fondazione Policlinico Universitario Agostino Gemelli IRCCS” in Rome, Italy, an innovative multidisciplinary model for the management of breast cancer, called ROME (Radiological and Oncoplastic Multidisciplinary Evaluation), has been developed. This innovative model integrates the expertise of multiple specialists into a unified, patient-centered approach. 

The ROME framework brings together radiologists, oncologists, surgeons, pathologists, oncoplastic specialists and psychologists to ensure a comprehensive assessment and personalized treatment plan for each patient. The ROME model begins with a thorough radiological assessment using advanced imaging techniques, followed by an oncoplastic assessment to identify surgical approaches that achieve optimal oncologic resection while maintaining or improving breast aesthetics.

**Figure 1:**
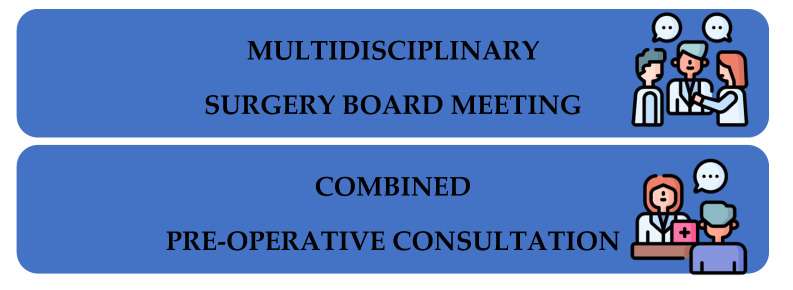

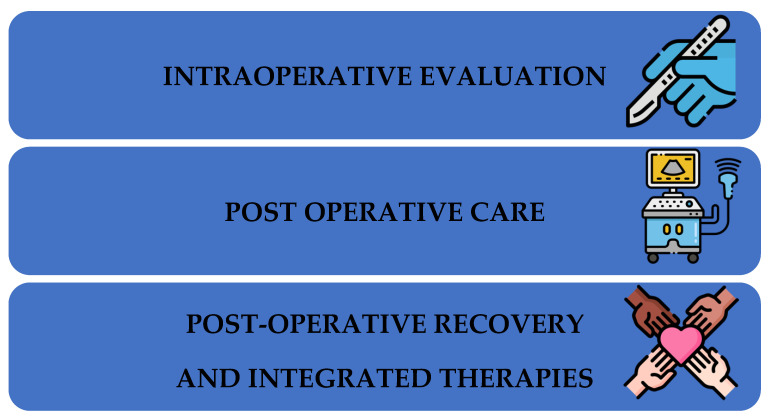
The five fundamental steps of ROME (Radiological and Oncoplastic Multidisciplinary Evaluation).

This combination of assessments allows the team to customize treatment strategies based on the patient’s individual clinical profile, including tumor characteristics, genetic factors, and aesthetic preferences. By integrating evidence-based guidelines with real-time multidisciplinary input, ROME ensures that treatment plans balance the need for effective cancer control with the desire for an optimal aesthetic outcome. The success of the ROME model has led to significant improvements in both oncologic and aesthetic outcomes, contributing to increased patient satisfaction and quality of life. In this article, we describe the ROME model in detail, analyzing the various phases of our diagnostic–therapeutic path and illustrating how this multidisciplinary, patient-centered approach, may represent a new standard for personalized breast cancer care in breast units (Figure 1).

## 2. Multidisciplinary Surgery Board Meeting

A multidisciplinary surgery board meeting is held weekly to allow all specialists to discuss diagnosis, staging, management and complementary therapies for new cases, complex patients and complications and employ evidence-based recommendations to construct an individual treatment plan. Additional cases requiring major or minor revisional surgery following complications are also discussed at this time. The multidisciplinary team reviews the diagnostic work-up, including personal history, pharmacological therapy, tumor characteristics, radiological examinations, previous breast surgery and systemic therapies and preoperative images of the patient to select the best surgical plan. Integrating the best clinical and scientific evidence, centered around consensus-based guidelines and EBM, is essential to develop personalized treatment plans [4]. 

**Figure 2:**
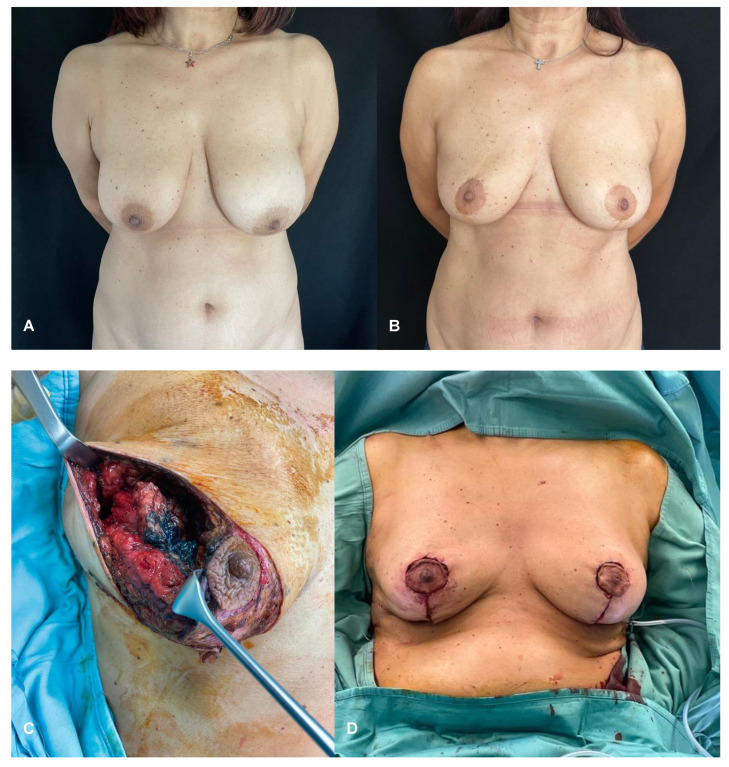
Patient with right breast cancer undergoing oncoplastic redcution mammaplasty (ORM) with J-scar. (**A**) Preoperative view. (**B**) Two-year postoperative view after adjuvant radiotherapy of the right breast. (**C**) Intraoperative view following right supero-external quadrantectomy with specimen weight of 80 g. (**D**) Immediate intraoperative result.

Tumor characteristics, such as size and location, tumoral resection, breast size, shape, and glandular density, previous breast surgery, and the patient’s desires are major determining factors in surgical planning. Patients with operable breast cancer may undergo breast-conserving surgery (BCS) or conservative mastectomy with immediate breast reconstruction (IBR) [5]. BCS has largely become the standard of care for low tumor-to-breast volume and early-stage breast cancer cases [6,7]. When <20% of the breast volume is removed, level I oncoplastic surgery (OPS) techniques are applied with the use of simple advancement or rotation flaps to fill the resected area [8]. Level II OPS is the most frequently indicated in breast cancer with large and/or ptotic breasts for which attempting a standard conserving surgery with safe margins may result in a breast deformity or the surgery being unsuccessful, a high tumor-to-breast volume ratio, and multifocal breast cancer [8]. Defects following BCS can be repaired using volume displacement or volume replacement techniques with contralateral symmetry surgery as appropriate [9]. OPS has extended the indications for BCS, and allows techniques from simple glandular reshaping to breast tissue mobilization without compromising the natural shape of the breast. Factors involved in selecting OPS are the tumor size-to-breast volume rate, tumor location, degree of mammary ptosis/macromastia and pre-existing breast asymmetry [8]. When 20–50% of the breast volume is resected in patients with large and ptotic breasts, level II OPS techniques are applied [10]. However, patients with small breasts undergo mastectomy to preserve breast aesthetics. The authors typically use volume displacement techniques, such as oncoplastic reduction mammaplasty (ORM) with contralateral symmetrization, to maintain breast aesthetics. With regard to therapeutic mammaplasty, we preferably apply J-scar mammaplasty technique over inverted T pattern mammaplasty as it guarantees a reduced complication rate [11]. OPS has not only proven to further expand breast conservative surgery with wider resection and lower recurrence rates, but has also been found to result in superior aesthetic results (Figure 2).

Common indications for nipple-sparing mastectomy (NSM) include a large tumor-to-breast ratio, an impossibility of obtaining oncologically safe margins with BCS or when it is impossible to guarantee adequate local control of the tumor, as well as in cases of multicentric disease, extensive ductal carcinoma in situ (DCIS) or substantial microcalcifcations, inherited high-risk gene mutations such as BRCA 1 and 2, contraindications to adjuvant radiotherapy, based on patient’s preference and for optimal aesthetic results; absolute contraindications for NSM include inflammatory breast cancer and locally advanced tumors involving the skin or nipple–areola complex (NAC) [12,13]. For those undergoing mastectomy, immediate breast reconstruction (IBR) is considered an oncologically safe technique for selected patients that permits enhanced quality of life and aesthetic outcomes.

Mastectomy flap thickness (MFT) is evaluated within digital mammography according to the Rancati score to determine the patients who may be candidates for prepectoral (PP) breast reconstruction [14]. We perform PP implant-based reconstruction, mainly Direct-To-Implant (DTI), in over 90% of conservative mastectomies, in accordance with the Italian data related to the Italian Senology Centers belonging to Senonetwork [15]. Thanks to the innovation of PP-DTI, this cost-effective technique demonstrates a reduction in operative time and complications related to submuscular breast implant placement, a reduction in contralateral breast symmetrization in unilateral cases, excellent aesthetic outcomes and superior quality of life [12,16,17]. 

**Figure 3:**
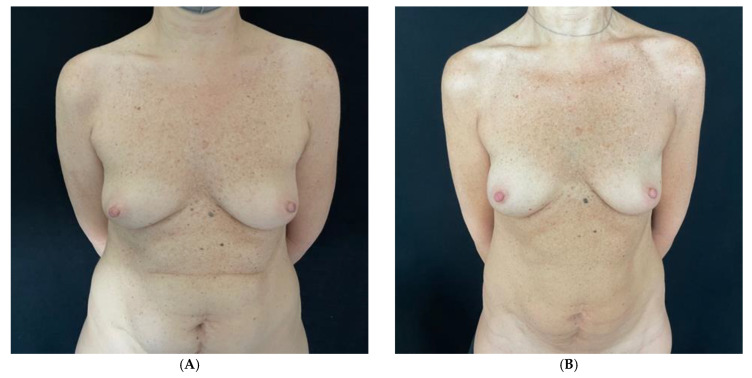

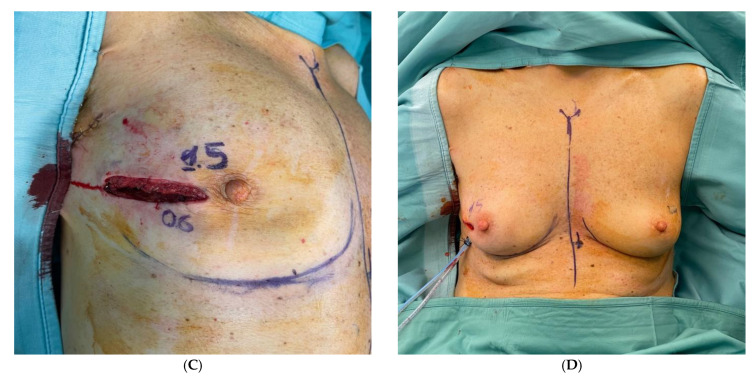
Patient undergoing right nipple-sparing mastectomy (NSM) and immediate prepectoral breast reconstruction. (**A**) Preoperative view. (**B**) Postoperative view after a 2-year follow-up. (**C**) Intraoperative view of the mastectomy flap thickness of the right breast at the incision site. (**D**) Immediate intraoperative view after prepectoral placement of short height, high projection, polyurethane-covered 115 cc anatomical implant.

**Figure 4:**
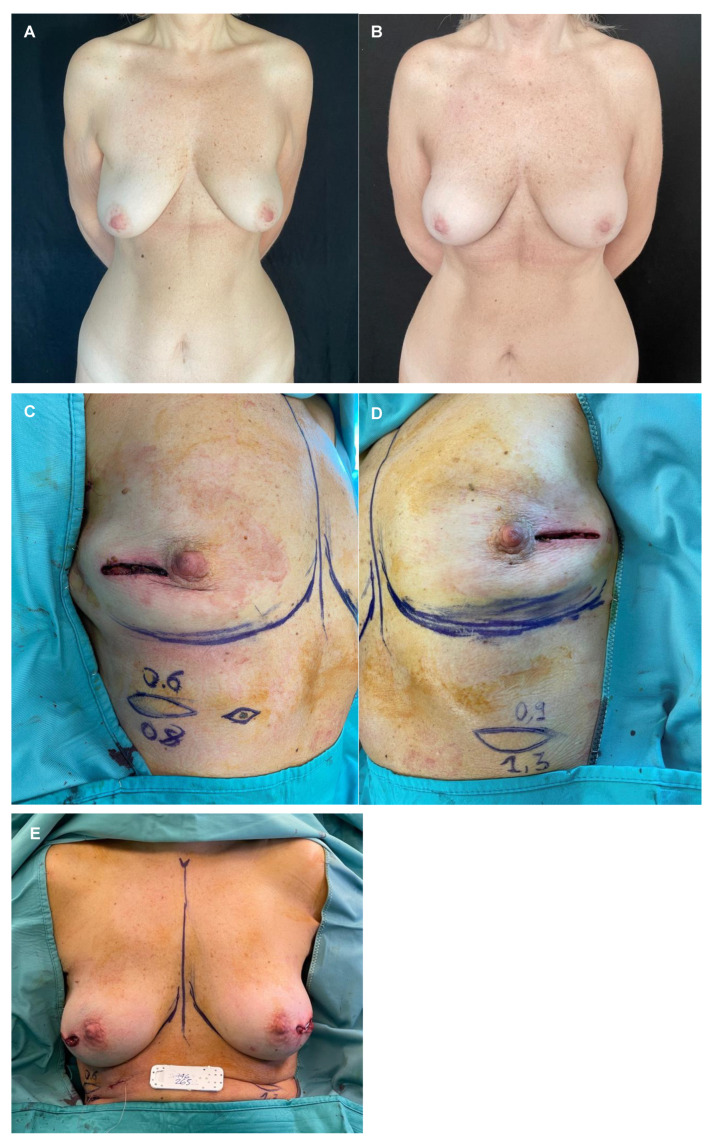
Patient with right breast cancer undergoing bilateral NSM due to BRCA mutation and immediate prepectoral breast reconstruction bilaterally. (**A**) Preoperative view with noticeable breast asymmetry. (**B**) Postoperative view after 3-year follow-up. (**C**,**D**) Intraoperative view of the thickness measurements of the mastectomy flaps of both breasts at the incision site. (**E**) Immediate intraoperative view after prepectoral placement of short height, high projection, polyurethane-covered 265 cc anatomical implants, bilaterally.

Patients with thick mastectomy flaps (Rancati scores of 2 and 3, i.e., MFT equal to or greater than 1 cm) undergo PP-DTI breast reconstruction. In the case of thin mastectomy flaps (MFT between 0.7 cm and 1 cm measured at the site of incision) and those well vascularized based on ICG angiography, patients undergo PP-DTI (Figure 3 and Figure 4). Patients with thin mastectomy flaps (0.5–0.7 mm measured at the site of incision) that are well vascularized at ICG angiography, preferably in bilateral cases, undergo renewed dual plane DTI breast reconstruction [18]. Thin patients with poorly vascularized mastectomy flaps at ICG angiography undergo submuscular–subfascial tissue expander placement [19].

Patients with very large and ptotic breasts who are poor candidates for NSM may undergo skin-reducing mastectomy (SRM) with J-scar patterns or inverted T patterns. PP DTI reconstruction is performed in cases of thick mastectomy flaps (Rancati score 2 and 3). Patients with a Rancati score of 1 undergo submuscular–subfascial tissue expander placement.

Patients who are obese or who are poor candidates for implant-based reconstruction may undergo Goldilocks mastectomy, a simple mastectomy without implant reconstruction that uses redundant mastectomy flap tissue alone to create a breast mound [20].

If the patient has a history of breast augmentation, a thorough evaluation is performed, and surgery is planned according to the oncological resection required and MFT. In case of BCS and abundant residual gland, the implants are removed, and the remaining gland is remodeled. In patients undergoing mastectomy, our preferred reconstruction is PP DTI (Rancati score 2 and 3), and submuscular DTI reconstruction in Rancati score 1 patients [21].

**Figure 5:**
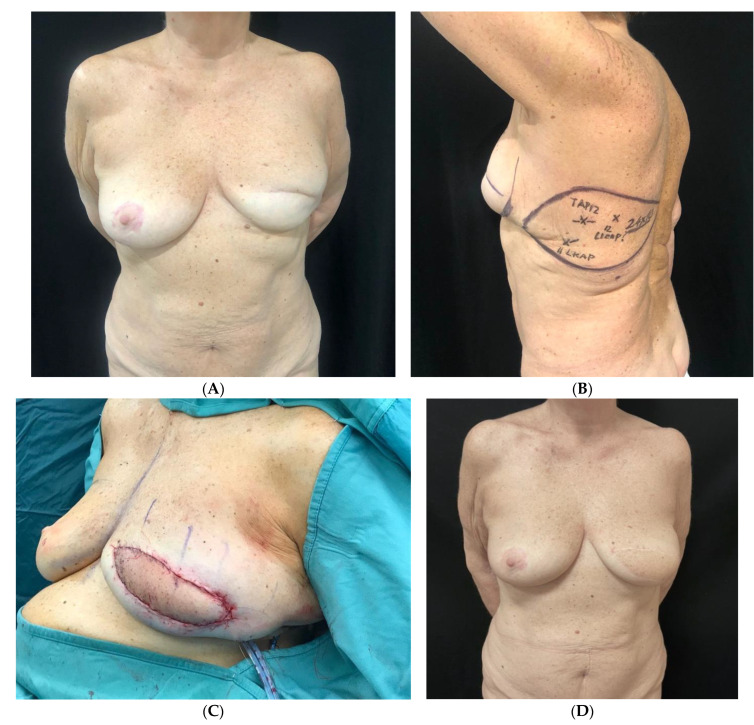
Patient planned for delayed left breast reconstruction with TDAP flap following previous skin sparing mastectomy (SSM) and submuscular tissue expander placement. (**A**) Preoperative view. (**B**) Preoperative markings of the skin paddle and TDAP perforators identified at ultrasound. (**C**) Intraoperative view following flap inset. (**D**) Postoperative view after 3-year follow-up.

**Figure 6:**
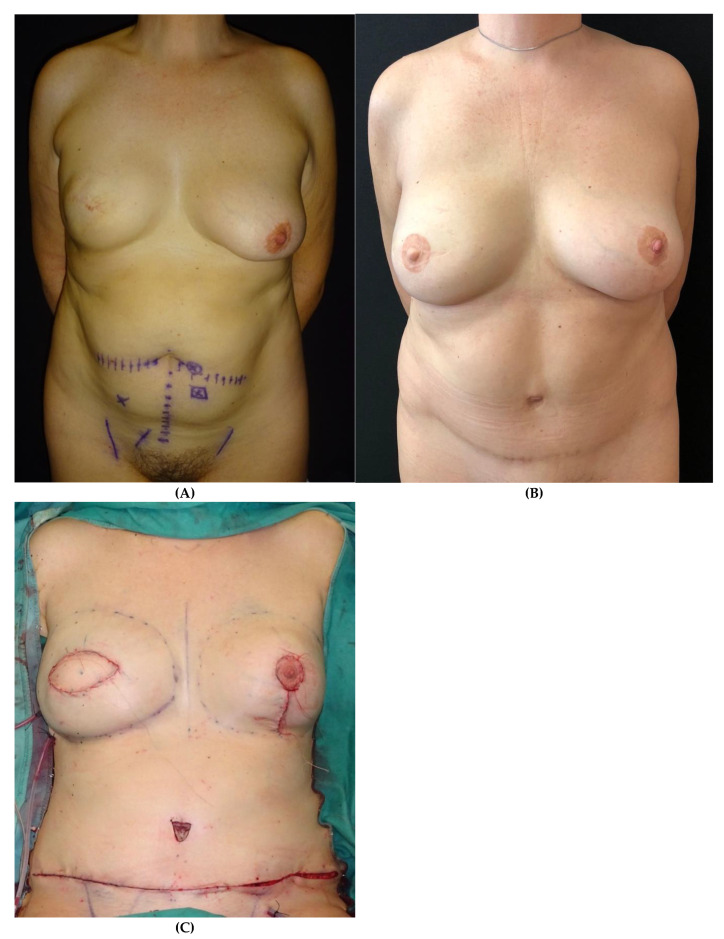
Patient who underwent delayed right breast reconstruction with the DIEP flap following previous skin-sparing mastectomy (SSM) and submuscular tissue expander placement. (**A**) Preoperative view. (**B**) Postoperative view after 2-year follow-up. (**C**) Intraoperative view following flap inset and contralateral mastopexy.

Generally, in patients who have undergone previous radiotherapy of the breast, as would be done for BCS and radiation treatment (RT), given the high incidence of complications, the authors opt for autologous-based reconstruction with flaps. Autologous breast reconstruction is planned at the joined consultation where the plastic surgeons, together with the breast surgeon, select the most indicated flap according to the patient’s somatotype, coagulative status, and personal wishes. All patients undergo a preoperative screening to identify asymptomatic or unidentified coagulation disorders or prothrombotic status. According to the literature, the DIEP flap is our gold standard, and the PAP flap is considered in patient without excess abdominal skin or previous abdominoplasty (Figure 5) [22,23]. In prothrombotic patients, a lateral intercostal artery perforator (LICAP) flap, a thoracodorsal artery perforator (TDAP) flap, or a pedicled latissimus dorsi (LD) flap is planned (Figure 6).

## 3. Combined Preoperative Consultation

At the time of the weekly surgery board meeting, a consultation with both the breast surgeon and plastic surgeon is scheduled in order to perform a joined assessment of the patient. Any additional appointments are scheduled at this time if deemed necessary. When possible, a multidisciplinary consultation is scheduled for the same day as the preoperative evaluation to limit patient discomfort in returning several times to the hospital. A preoperative psychological evaluation is also provided for all patients at this time.

The plastic surgeon and breast surgeon together perform a detailed evaluation of the patient that includes:

- Demographic variables (age, weight, height, BMI) and medical history, current pharmacological therapy, type of breast cancer and location, neoadjuvant chemotherapy, previous radiotherapy, and previous aesthetic or reconstructive breast surgery.
- Clinical breast and chest examination to determine breast shape and volume, the presence of breast anomalies or chest malformations, the presence of scars from previous breast surgery, signs of previous radiotherapy including fibrosis or skin alterations, presence of implants from previous breast augmentation, collect linear breast measurements (sternal notch-to-nipple distance, nipple-to-inframammary fold distance, nipple-to-nipple distance and chest, waist and hip circumference). The plastic surgeon and the breast surgeon decide together when to perform an autologous or implant-based reconstruction. In case of implant-based reconstruction, the position of the implant (whether prepectoral or retropectoral) is chosen based on mammograms and clinical evaluation, including MFT, and based on the Rancati score for the classification of breast tissue coverage (BTCC) [14].
- Skin incision is also planned. In patients undergoing mastectomy and IBR, the skin incision may be radial lateral, at the inframammary fold, or planned as a skin-reducing mastectomy (SRM) in case of very large and ptotic breasts. The radial incision provides the best access for performing mastectomy while also maintaining maximal vascularization of the NAC [24,25]. In case of nipple resection for tumor infiltration after intraoperative frozen section, the radial incision may be extended to include the NAC. The mastectomy may also be performed through the IMF incision when the breast is small and without ptosis. We prefer the lateral IMF approach, as it provides adequate exposure, eliminates visible scars on the anterior surface of the breast and preserves the anterior intercostal artery perforator (AICAP), an important neurovascular pedicle for the NAC [26]. Previous breast scars represent another skin access, preferred for avoiding devascularization of the NAC, particularly in patients who have previously undergone breast reduction surgery.
- Photographic documentation of the patient’s breasts in the frontal, oblique and lateral projections.
- Review and discussion of the personalized surgical plan and postoperative indications with the patient and their family.

Patients undergoing level II OPS with displacement techniques, such as a oncoplastic reduction mammaplasty (ORM), are marked bilaterally for a J-scar mammaplasty by the plastic surgeon following tumor localization with preoperative breast ultrasound and/or mammogram [11].

## 4. Intraoperative Evaluation

In the case of ORM with the J-scar mammaplasty, partial breast resection is performed by the breast surgeon through the planned incisions marked by the plastic surgeon. Following tumor resection, the plastic surgeon appropriately mobilizes the residual parenchyma by creating a dermo-glandular flap to fill the defect. The vascularization of the flap may be superomedial for defects of the external breast quadrants, superolateral for defects of the inferointernal quadrants, and inferior for defects of the superior quadrants [27]. The resected gland is weighted to perform an analogous resection of the inferior quadrants of the contralateral breast reduction and maintain breast symmetry.

The surgical treatment of axillary lymph nodes is performed according to the latest NCCN guidelines [28].

For patients undergoing mastectomy, the mastectomy flap thickness is intraoperatively assessed at the margins of the surgical incision. We perform an intraoperative ICG angiography on all patients to determine whether or not PP-DTI is feasible and if the NAC may be preserved. If the mastectomy flaps are well vascularized, PP-DTI is deemed appropriate and performed. The mastectomy specimen is weighted and, depending on its weight, implant sizers are used to select the breast implant that best fills the prepectoral pocket. The volume of the breast implant is usually equal to or 20% greater than the weight of the mastectomy specimen. Patients undergo skin-sparing mastectomy (SSM) in case of tumor involvement of the NAC. In case of implant-based IBR, the implant is slightly smaller than the mastectomy specimen. A contralateral breast reduction is usually performed to obtain symmetry.

Nowadays, in our center, all patients receive polyurethane-coated, anatomical, short or medium-height breast implants. We prefer polyurethane-coated implants for the stability provided and the integration into the surrounding tissues which reduces the dead space and the incidence of postoperative seromas [29,30].

In cases of suboptimal vascularization of the mastectomy flaps, a submuscular–subfascial tissue expander is placed [19].

Immediately upon skin closure, a single-use negative pressure wound therapy (sNPWT) care system is applied to the surgical wound and maintained for 7 days postoperatively. The sNPWT care system is systematically used in high-risk patients such as smokers, patients with obesity, and patients with thin mastectomy flaps undergoing PP or renewed dual plane DTI.

## 5. Postoperative Care

Proper patient education is fundamental to avoid early postoperative complications. At the time of hospital discharge, usually after 2 days, every patient is instructed on how to perform surgical drain care every 24 h and maintain arm and shoulder rest to avoid tension on surgical incisions and fluid accumulation. Patients without sNPWT are also instructed on how to perform proper wound care.

Postoperative check-ups are performed at the out-patient breast unit clinic at the FPG. At the first scheduled postoperative check-up on postoperative day 7, the sNPWT care system is removed from the surgical wound. Upon dressing removal, we evaluate the surgical wound and determine if there is any delay in wound healing. In such cases, patients undergo surgical wound revision in a dedicated outpatient clinic setting. Patients are instructed to maintain the surgical wounds cleanliness and perform wound dressing changes every 24 h. Surgical drains are assessed at every postoperative evaluation and removed when fluid collection is less than 20 mL and stable in the preceding 24 h.

Additionally, attentive postoperative care following breast surgery and reconstruction is important to identify the early signs of complications. Proper wound and surgical drain management are fundamental for a successful implant-based breast reconstruction. The addition of breast ultrasound (US) may identify fluid collections and reduce the risk of infection, would dehiscence and implant loss [31].

Breast US is a vital tool for breast surgery. US is useful in the initial evaluation of patients presenting with signs and symptoms of fluid accumulation in the axillary cavity or in the implant pocket [32]. During postoperative check-ups, patients routinely undergo breast USs with the aid of a color-coded duplex sonography 2–3 days after surgical removal of the drain to check that there is no fluid around the implant. US evaluation is certainly performed if signs or symptoms of clinical or subclinical infection are present, or in case of a sudden increase in breast volume [33].

In the case of significant fluid accumulation in the axillary cavity, a US-guided needle aspiration may be performed [34]. After aspiration, a compressive dressing is applied to the area of the aspirated area to reduce further fluid accumulation. The patient is re-evaluated 3–5 days following fluid aspiration. A fluid aspiration may be repeated in cases of large fluid accumulations. Repeated aspirations are avoided if the fluid accumulation is significantly smaller.

In cases of fluid collection around the implant, the size and characteristics are determined through US evaluation. If the fluid collection is small, less than 1 cm in thickness, hyperechoic, adherent to the implant surface, or not associated with signs or symptoms of local or systemic infection, fluid aspiration is avoided, and the patient is monitored by US for the following week. If the fluid collection is >1 cm in thickness, anechoic, or associated with signs of local or systemic infection, US-guided needle aspiration is performed, followed by fluid analysis with culture and Gram-stain. Anti-inflammatory therapy and a compressive dressing are applied if no signs of local or systemic infection are present. A broad-spectrum antibiotic therapy is prescribed for patients with signs and symptoms that suggest an infection, and is continued until the causative agent is revealed, then the appropriate antibiotic is selected. Blood tests, including a complete blood count and indicators of inflammation, are prescribed prior to and throughout the course of antibiotic therapy. Patients are re-evaluated 3 to 5 days later. In the absence of fluid accumulation or the presence of small fluid accumulations, no aspiration is performed. In the presence of recurrent and symptomatic fluid accumulations, US-guided needle aspiration is repeated, and the treatment proceeds as previously described.

If, after surgical drain removal, no fluid accumulations have occurred, patients are instructed to gradually begin arm and shoulder movement to regain mobility and functionality. Simple exercises may be performed a few times a day for 5–10 min. In cases of limited range of motion, patients are advised to undergo physical rehabilitation with a physiotherapist [34].

After complete wound healing, all patients are prescribed a silicone-based gel to improve final scar appearance [35]. A postoperative evaluation is planned at 3, 6 and 12 months.

In the case of adjuvant radiotherapy, secondary surgical interventions, such as lipofilling, are discussed with the patient.

## 6. Postoperative Functional Recovery Through Integrated Therapies

Postoperative recovery following breast cancer surgery is a process that requires more than just physical healing; it also involves emotional, psychological and aesthetic rehabilitation [36]. In the context of the ROME model, the inclusion of integrated therapies plays a pivotal role in enhancing functional recovery, preventing complications such as lymphedema and improving aesthetic outcomes.

- Psychological support in postoperative care

Psychological well-being is a cornerstone of the recovery process after breast cancer surgery. The emotional toll of the diagnosis and treatment can significantly impact patients’ mental health, self-esteem and body image [37]. Within the ROME framework, psychological support is integrated as part of the multidisciplinary care approach, providing patients with professional counseling and therapeutic support to navigate the emotional complexities of their recovery journey.

Psychological counseling helps patients address feelings of anxiety, depression and body image issues that may arise post-surgery [38]. Support groups and individual therapy sessions are offered to foster emotional resilience, encourage coping strategies and enhance overall mental well-being. This holistic support system not only accelerates emotional healing but also improves physical recovery by empowering patients to take an active role in their rehabilitation and by enhancing their overall quality of life.

- Physiotherapy for functional recovery and lymphedema prevention

Physiotherapy is an essential part of postoperative care in breast cancer patients, focusing on both functional recovery and the prevention of complications like lymphedema [39,40]. Following surgery, patients often experience limitations in arm mobility, strength and range of motion that may impair daily activities and prolong the recovery process. A tailored physiotherapy program designed to restore upper body function is crucial to regain mobility and independence.

Breast cancer-related lymphedema is a concern in patients undergoing lymph node dissection, particularly in patients with additional risk factors such as obesity and adjuvant radiotherapy on the ipsilateral side [41,42,43]. Lymphedema leads to fluid retention, swelling and discomfort in the arm that can significantly impact quality of life. In the ROME model, physiotherapists work proactively to prevent the onset of lymphedema through manual lymphatic drainage and specific exercises to promote lymphatic flow and education on self-care techniques. These methods help patients reduce the risk of lymphedema, improve arm function and prevent long-term complications [44].

- Endermology for Aesthetic Outcomes

Endermology, a non-invasive treatment using mechanical massage, is increasingly being employed in postoperative breast cancer care to enhance aesthetic outcomes. The procedure stimulates the skin and underlying tissue, promoting lymphatic drainage, improving blood circulation and encouraging tissue regeneration [45]. For breast cancer patients, endermology offers significant benefits in improving skin elasticity, reducing the appearance of scars and enhancing overall breast aesthetics after surgery.

Endermology can be particularly useful for patients undergoing breast-conserving surgery and autologous reconstruction, as it helps to refine and smooth the contours of the breast, contributing to more natural-looking results. In conjunction with other therapies, such as physiotherapy and psychological support, endermology helps restore the aesthetic appearance of the breast, addressing both functional and emotional recovery needs.

## 7. Conclusions

**Figure 7:**
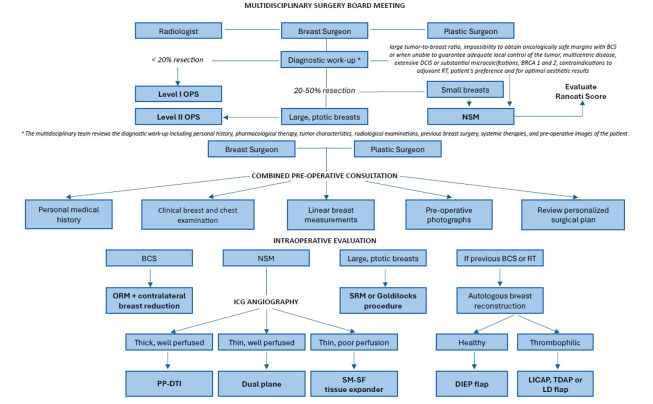

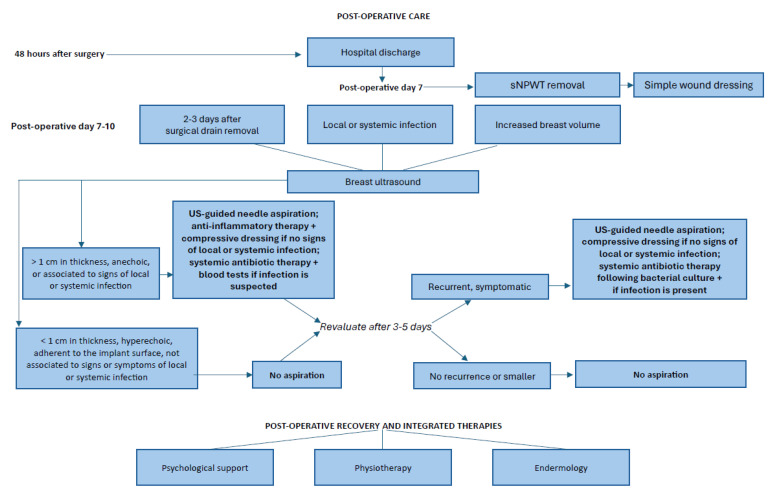
ROME (Radiological and Oncoplastic Multidisciplinary Evaluation) Flowchart.

The ROME (Radiological and Oncoplastic Multidisciplinary Evaluation) model (Figure 7) represents a major step forward in the personalized care of breast cancer patients. By bringing together the expertise of multiple specialists within a collaborative, evidence-based framework, ROME ensures that treatment plans are meticulously tailored to meet each patient’s unique clinical and aesthetic needs.

The success of this model is based on its comprehensive approach, combining detailed radiological assessments, oncoplastic evaluations and continuous multidisciplinary input throughout every stage of the treatment process—from preoperative planning to postoperative care through integrated therapies. The multidisciplinary surgery board meetings and combined preoperative consultations play a crucial role in developing individualized treatment strategies that effectively balance oncological outcomes with the preservation of breast aesthetics.

This collaborative approach continues into the operating room and postoperative care, ensuring the highest standard of care and the best possible outcomes in both oncological control and cosmetic results. The ROME model may not only enhance patient satisfaction but also contribute to a better quality of life for breast cancer patients. The integration of clinical, radiological, and aesthetic considerations through a patient-centered approach is setting a new benchmark for breast cancer care. Looking forward, ROME represents a pioneering model for personalized breast cancer surgery and reinforces the need for a holistic, multidisciplinary approach to oncology, marking a new era in the standard of care for breast cancer patients.

## Data Availability

No new data were created or analyzed in this study. Data sharing is not applicable to this article.
